# Violation of the Unity Assumption Disrupts Temporal Ventriloquism Effect in Starlings

**DOI:** 10.3389/fpsyg.2018.01386

**Published:** 2018-08-14

**Authors:** Gesa Feenders, Georg M. Klump

**Affiliations:** Cluster of Excellence Hearing4all, Animal Physiology and Behaviour Group, Department of Neuroscience, School of Medicine and Health Sciences, University of Oldenburg, Oldenburg, Germany

**Keywords:** multisensory integration, avian models, unity assumption, temporal order judgment, visual processing, auditory processing

## Abstract

When stimuli from different sensory modalities are received, they may be combined by the brain to form a multisensory percept. One key mechanism for multisensory binding is the unity assumption under which multisensory stimuli that share certain physical properties like temporal and/or spatial correspondence are grouped together as deriving from one object. In humans, evidence for a role of the unity assumption has been found in spatial tasks and also in temporal tasks using stimuli that share physical properties (speech-related stimuli, musical and synesthetically congruent stimuli). In our study, we investigate the role of the unity assumption in an animal model in a temporal order judgment task. When subjects are asked to indicate which of two spatially separated visual stimuli appeared first in time, performance improves when the visual stimuli are paired (in time) with spatially non-informative acoustic cues, a phenomenon known as the temporal ventriloquism effect. Here, we show that European starlings perform better when one singleton acoustic cue is paired with the first visual stimulus as compared to pairing with the second visual stimulus. This shows, in combination with our previous study, that a non-informative singleton acoustic cue, when temporally paired with the first visual stimulus, triggers alerting while, when temporally pairing with the second visual stimulus, it prevents a temporal ventriloquism effect because the unity assumption is violated. Thus, the unity assumption influences sensory perception not only in humans but also in an animal model. The importance of the unity assumption in this task supports the idea that the temporal ventriloquism effect, similar to the spatial ventriloquism effect, is based on multisensory binding and integration but not on alerting effects.

## Introduction

In our brain, stimuli from all our senses work in concert to evoke percepts of our environment. For this, stimuli with similar temporal and spatial properties may be grouped as originating from one source and can thus be better segregated from other stimulus sources by multisensory integration (reviewed in Stein and Stanford, 2008).

In general, if two stimuli from different sensory modalities are received close in time, they can have two effects. On the one hand, if the earlier stimulus is a non-target stimulus (i.e., irrelevant for the actual task) this may induce neuronal facilitation that will evoke a faster reaction to the following target stimulus, an alerting effect that can also lead to the “prior entry effect” (Spence et al., 2001; Schneider and Bavelier, 2003; Santangelo and Spence, 2009; Barrett and Krumbholz, 2012; for a review see Spence and Parise, 2010). On the other hand, if the unisensory stimuli arrive at a multisensory neuron within a critical time window of integration (which is usually defined by the leading sense, Meredith et al., 1987; Diederich and Colonius, 2004, 2015; Powers et al., 2009) this may evoke an integrated or combined response in the multisensory neuron which is widely discussed as multisensory integration (as reviewed in Stein and Stanford, 2008; Chen and Vroomen, 2013).

In the temporal order judgment (TOJ) task, researchers can manipulate stimuli to either act as alerting signals or potentially evoke multisensory integration. In the vision-based version of this task, the subject has to judge which of two visual stimuli (for example, the upper or lower light) appeared first. If non-informative acoustic cues (for example, short noise bursts from a central position) are added to the visual stimuli, the performance may change: one acoustic cue (A1) temporally leading the first visual stimulus (V1) and a second acoustic cue (A2) temporally following the second visual stimulus (V2) will yield better performance than acoustic cues presented simultaneous to the visual stimuli as has been shown in humans (Morein-Zamir et al., 2003) and in European starlings (*Sturnus vulgaris*, Feenders et al., 2017). This effect is known as the temporal ventriloquism effect (henceforth TVE, compare with the spatial ventriloquism effect, see Chen and Vroomen, 2013). Morein-Zamir et al. (2003) further demonstrated that the TVE was evoked when A2 was lagging V2 with A1 being presented simultaneous to V1, but when A1 was leading V1 and A2 was simultaneous to V2 there was no TVE. Thus, they argue that the TVE is driven by the lagging acoustic cue. It is important to note that the time offset between the acoustic and visual stimuli has to be chosen carefully to match the size of the temporal binding window which is task-specific (Meredith et al., 1987; Vatakis and Spence, 2010; Vroomen and Keetels, 2010): this window is larger for visually guided than for acoustically guided stimuli (due to extended processing times of the visual as compared to the auditory system), thus a time offset which matches the size of the temporal binding window of both modalities is necessary (in case of audio-visual experiments this is usually around 75–100 ms).

Combining different sensory modalities in the processing of stimuli is important for communication in many animals including birds (reviewed in Rowe, 1999; Partan and Marler, 2005). For example, audio-visual signals are synchronized during courtship behavior (Partan et al., 2005; Ullrich et al., 2016; for a review on birdsong and singing behavior, see Williams, 2004) and visual stimuli facilitate song learning in nightingales, *Luscinia megarhynchos* (Hultsch et al., 1999). In European starlings, the song structure is temporally precisely synchronized with specific wing movements during singing (Böhner and Veit, 1993) and song acquisition is compromised when the birds experience only tape-recordings instead of live tutors providing audio-visual signals (Chaiken et al., 1993). The ethological significance of audio-visual binding makes the starling a well-suited animal model to investigate the TVE.

In our previous study on starlings, we established the TVE evoked by a visual TOJ task with acoustic cues flanking the visual stimuli (Feenders et al., 2017): the birds’ performance improved when the first acoustic cue (a 5 ms broadband noise) was presented prior to the first visual stimulus (a small LED turned on) and the second acoustic cue presented after the second visual stimulus as compared to acoustic cues presented simultaneous to the visual stimuli. When using two acoustic cues with asymmetric time offsets (i.e., one cue synchronous and the other with a time offset), the TVE was best evoked with a lagging second acoustic cue but not with a leading first acoustic cue. We could further show that a singleton acoustic cue improved performance when preceding the first visual stimulus and thus serving as an alerting signal. One major difference between these test paradigms is the consistency of acoustic-visual pairing. The presentation of two acoustic and two visual stimuli can evoke intra-modal Gestalt grouping in both the visual and the auditory domain (i.e., the Gestalt of a pair of stimuli) as the observer experiences multiple repetitions of the test stimuli throughout the session. If those pairs are furthermore presented in close temporal proximity to each other, the observer may assume that the visual and the acoustic stimulus pair belong together, the so-called “unity assumption” or “rule of unity” (reviewed by Welch and Warren, 1980; Vatakis and Spence, 2007; Chen and Spence, 2017). This unity assumption will then enable cross-modal binding and multisensory integration (see Spence, 2015 for a review of Gestalt and multisensory binding). In contrast, with a singleton leading acoustic cue, such visual-acoustic pairing is not possible due to a lack of temporal correspondence and thus no assumption of unity. Consequently, the leading acoustic cue may activate the alerting system rather than multisensory integration. One important question remains: if, as argued above, the TVE is driven by an acoustic cue presented after the second visual stimulus, would this also hold for a singleton acoustic cue violating the unity assumption? In other words, how important is the unity assumption for the TVE in the TOJ task? This is an important issue as pointed out by Chen and Spence (2017), because the unity assumption holds for spatial ventriloquism effects while its role in temporal ventriloquism effects seems limited. The unity effect in temporal ventriloquism paradigms has been reported for speech-related stimuli but not for non-speech stimuli (Vatakis and Spence, 2007, 2008; Vatakis et al., 2008) and for synesthetically congruent stimuli (i.e., a high-pitched sound is combined with a small visual stimulus and a low-pitched sound is combined with a large visual stimulus; Parise and Spence, 2008, 2009) and musical stimuli (Petrini et al., 2009; Chuen and Schutz, 2016). We investigated the question whether the unity assumption also applies to an animal model by comparing the performance of starlings in a vision-based TOJ task: a singleton acoustic cue was paired with either the first or the second visual stimulus and, in addition, the acoustic cue was presented either synchronous to the visual stimulus or with a short time offset producing a leading sound or a trailing sound. We expect a leading acoustic cue to have an alerting effect and thus to improve performance in comparison to the synchronous presentation. A trailing acoustic cue should evoke the TVE (with respect to the synchronous presentation) if the TVE is independent of the unity assumption. However, if the TVE requires compliance with the unity assumption to enable intra-modal perceptual grouping and cross-modal binding, we expect a singleton trailing acoustic cue to have no enhancing effect with respect to the synchronous stimulus presentation.

## Materials and Methods

### Subjects and Experimental Set-Up

Four wild-caught European starlings (males P and S, females I and M), age 3 years or older, participated in this study. They were housed indoors (15 h:9 h LD cycle, roughly 20–22°C room temperature) in individual cages with visual and acoustic contact to each other (for further details of the housing, see Feenders et al., 2017). The birds were provided with a restricted amount of food that was adjusted daily to keep them on average at 90% free-feeding weight in order to keep them motivated during testing.

All four birds had participated in a previous study (Feenders et al., 2017) using the same experimental set-up: a 50 cm-wide wire-mesh test cage was placed in a double-wall soundproof chamber (1200A series, Industrial Acoustics Company, United Kingdom). The chamber was illuminated homogenously by LEDs at the ceiling (Barthelme LEDlight flex 14, Germany) and the rear (Paulmann, Germany) of the chamber. An additional LED (Paulmann, Germany) served as a reward light (see below). The test cage was fitted with a start perch and two target perches at 210 mm distance and 25° to the left and right to the start perch. Each perch was fitted with light barriers (Conrad Electronics, Germany) in order to track the bird’s position. The bird was constantly observed via web cam (QuickCam Pro 9000, Logitech, Switzerland; 15 frames per second) mounted above the test cage. This study was carried out in accordance with the recommendations of EU Directive 2010/63/EU on the protection of animals used for scientific purposes. The experiments with the starlings were approved by the Niedersächsisches Landesamt für Verbraucherschutz und Lebensmittelsicherheit, Germany.

### Stimuli

Two SMD-LEDs (dominant wavelength 605 nm, Avago HSML-C150, Broadcom Ltd., CA, United States) were mounted at the rear of the test cage 22.5° to the left and the right of the start perch at a height matching the bird’s head. One loudspeaker (Vifa XT25TG30-04, ASE, Germany), connected to an amplifier (RMB-1048, Rotel, Japan), was mounted centrally (0°) 3 cm lower than the LEDs and produced a band-limited noise burst (1–4 kHz, 5 ms duration) of 80 dB SPL peak to achieve a perceived level of about 60 dB (see Klump and Maier, 1990). All electronic devices were controlled by two real-time processors (RP2 and RX6, Tucker Davis Technologies, Alachua, FL, United States) connected to a Linux-operated computer.

**FIGURE 1:**
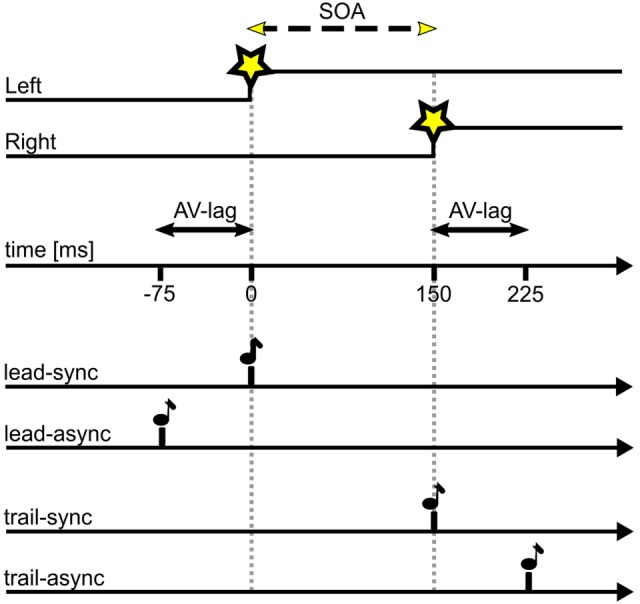
Timing of stimuli as presented in this study. SOA of variable duration as indicated by the dashed line, in this example SOA = 150 ms with left LED first; AV-lag of 75 ms. Star symbol, LED onset; note symbol, acoustic stimulus. A test trial consists of the visual stimulus (upper two traces) presented together with one of the four acoustic stimuli (lower four traces).

For the TOJ task, the two LEDs (V1, V2) were turned on with a stimulus-onset asynchrony (SOA) of either 25, 50, 75, 100, or 200 ms, either left or right LED leading. The acoustic cue (A) was presented with a simultaneous onset either to V1 (lead-sync: V1&A – SOA – V2) or V2 (trail-sync: V1 – SOA – V2&A) or with an audio-visual lag time (AV-lag) of 75 ms either leading V1 (lead-async: A – AV-lag – V1 – SOA – V2) or trailing V2 (trail-async: V1 – SOA – V2 – AV-lag – A). **Figure 1** illustrates the different conditions. Each bird was tested in one session per day, 5–6 days per week. In each session, the acoustic cue was either paired with V1 (leading) or with V2 (trailing) and comprised 10 warm-up trials of the synchronous condition (lead-sync or trail-sync) and 200 ms SOA (left or right leading) followed by 100 test trials. The warm-up trials allowed the highly trained birds to adapt to the test condition of the session. The test trials were organized in five blocks of 20 different test conditions (10 SOAs, two experimental conditions sync or async) in randomized order. Three “leading” sessions (including the test conditions lead-sync and lead-async) were followed by three “trailing” sessions (including the test conditions trail-sync and trail-async) and this scheme repeated until nine valid sessions (see below) of each type had been collected. This scheme was a compromise between avoiding a learning effect and allowing the birds to adjust to the leading or trailing test scheme.

### General Procedure

Once the bird sat on the start perch for 2–6 s, the stimuli were presented and the bird had to respond within 5 s by hopping either to the left or the right target perch indicating which LED was turned on first. In case of a correct reaction (hit) a reward was provided (a piece of cooked meal worm, *Tenebrio molitor*, Zoo Med, CA, United States, or a flour-based food pellet, own production). If the bird responded to the incorrect side (wrong) or not at all (miss), a 5s-blackout was introduced before the next trial started. The LEDs were turned off as soon as the bird made a response or the 5s-report phase elapsed, whichever occurred first.

### Data Analysis

For the analyses, we combined data from nine valid sessions as only five repetitions per individual test condition and SOA were obtained in a single session, resulting in hit rates with little statistical validity. All analyses were based on those nine sessions selected by applying the following criterion: For each session, the proportion of correct choices (henceforth hit rate) in test trials with SOAs of 100 ms and 200 ms and across experimental conditions was calculated and the session selected if the hit rate was significantly greater than chance (0.5%, validated by a binomial test; we expected performance above chance for trials with long SOAs as this should be easy for the bird). The hit rate was based on counts of hits and wrongs, excluding missed trials as these are difficult to interpret and classify as correct or incorrect. Bird S did not reach criterion in the trailing sessions but in the leading sessions. It is obvious that this bird had great difficulties solving the task with a trailing acoustic cue while having no problems in the leading sessions. Thus, for this bird we chose to include nine sessions of the trailing type in the analysis despite not meeting the criterion. Sessions of the leading type were chosen according to the above criterion.

First, to visualize the data for each subject, the proportion of responses to the right was plotted against the SOAs and a logistic function was fitted for each test condition to obtain a psychometric function (PMF; Matlab, Mathworks, Inc., MA, United States).

Second, the actual hit rate was analyzed: we expect stimuli that evoke the temporal ventriloquism or alerting effects to result in higher hit rates reflecting higher sensitivities. Within each subject, the data were combined across sides (to balance for side bias) and rank-transformed (Meddis, 1984) across conditions (due to the small sample size, normally distributed data are difficult to identify), followed by RM ANOVA to test for effects of order (lead or trail) and timing (sync or async).

Third, we investigated the effect of the test conditions on the number of missed trials because live observations during the experiments hinted at a relation between missed trials and difficulty of the trial. Namely, the birds sometimes appeared to withhold a response once the full set of stimuli had been perceived. Thus, the number of misses per condition was summed up across all SOAs and sessions (each bird received the same number of trials) and a RM ANOVA was performed to analyze for effects of order and timing.

All statistical calculations were implemented with R-project^1^.

## Results

**FIGURE 2:**
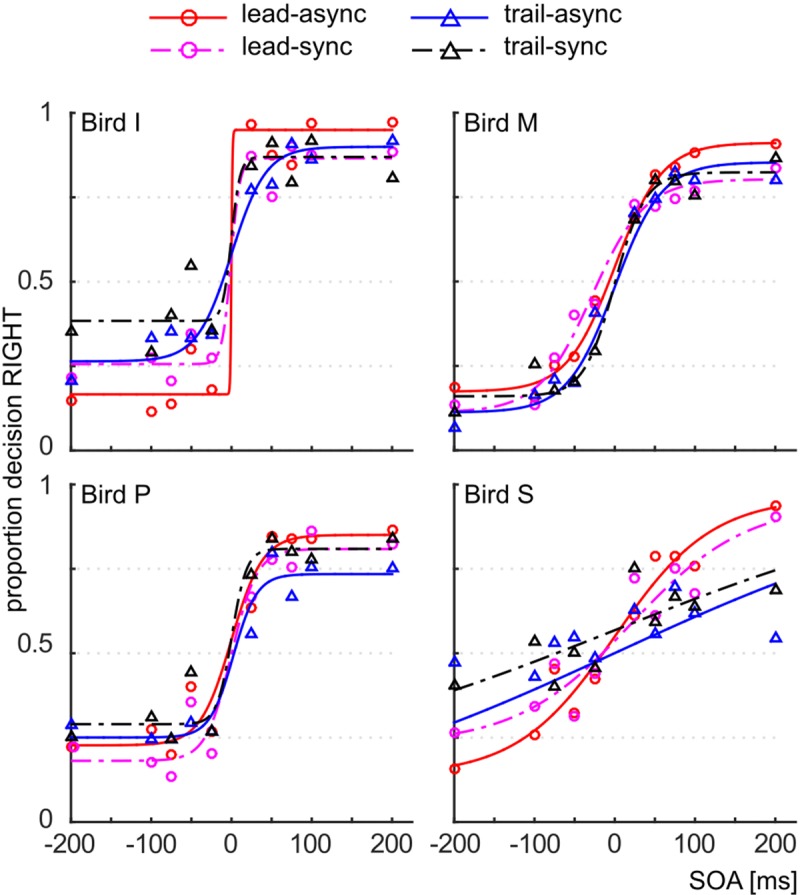
Correct responses. Proportion decision to the right as a function of the SOA, for the different test conditions. Data are shown as symbols, the fitted PMFs as line graphs. Red circle and solid line: lead-async; magenta circle and dashed line: lead-sync; blue triangle and solid line: trail-async; black circle and dashed line: trail-sync.

**FIGURE 3:**
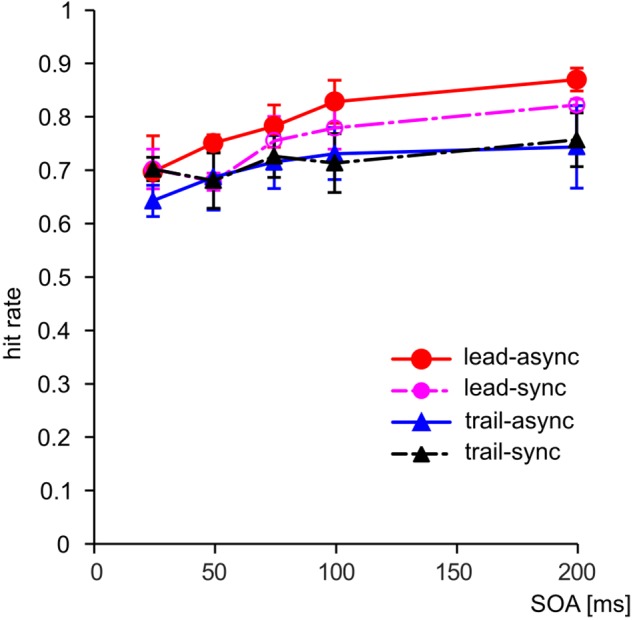
The hit rate combined across sides (positive/negative SOAs) as a function of SOA. Error bars: Standard error of the mean. Color coding as in **Figure 2**.

We asked whether violation of the unity assumption impairs performance in a vision-based TOJ task. The performance of the subjects for each test condition is shown as PMFs in **Figure 2**: the lead-async condition evoked best performance in all four birds, i.e., the steepest PMF slopes with low asymptotes for negative SOAs and high asymptotes for positive SOAs, while the trailing conditions (both trail-async and trail-sync) resulted in lower performance. We analyzed the ranked hit rate to test whether the performance is differently affected by order (leading versus trailing sound) and by timing (synchronous versus asynchronous sound; **Figure 3** shows the mean hit rate prior to rank transformation). We found a significant effect of order, *F*(1,57) = 17.67, *P* < 0.0001, and SOA, *F*(4,57) = 9.28, *P* < 0.0001, but no effect of timing, *F*(1,57) = 0.42, *P* = 0.519, or any interaction, *P* > 0.183. **Figure 3** shows a reduced performance for trailing conditions as compared to leading conditions.

A separate analysis of the data from the leading condition revealed a significant effect of timing, *F*(1,27) = 4.87, *P* = 0.036, and SOA, *F*(4,27) = 9.38, *P* < 0.001, but no interaction, *F*(4,27) = 0.44, *P* = 0.777. Thus, a leading, asynchronous acoustic cue evoked better performance than an acoustic cue presented synchronous with the first visual stimulus. This is in accordance with our previous finding (Feenders et al., 2017). For the trailing condition, there was no significant effect of either SOA, *F*(4,27) = 2.67, *P* = 0.054, timing, *F*(1,27) = 0.33, *P* = 0.570, or interaction, *F*(4,27) = 1.42, *P* = 0.253. This shows that a singleton trailing acoustic cue does not evoke the TVE.

**FIGURE 4:**
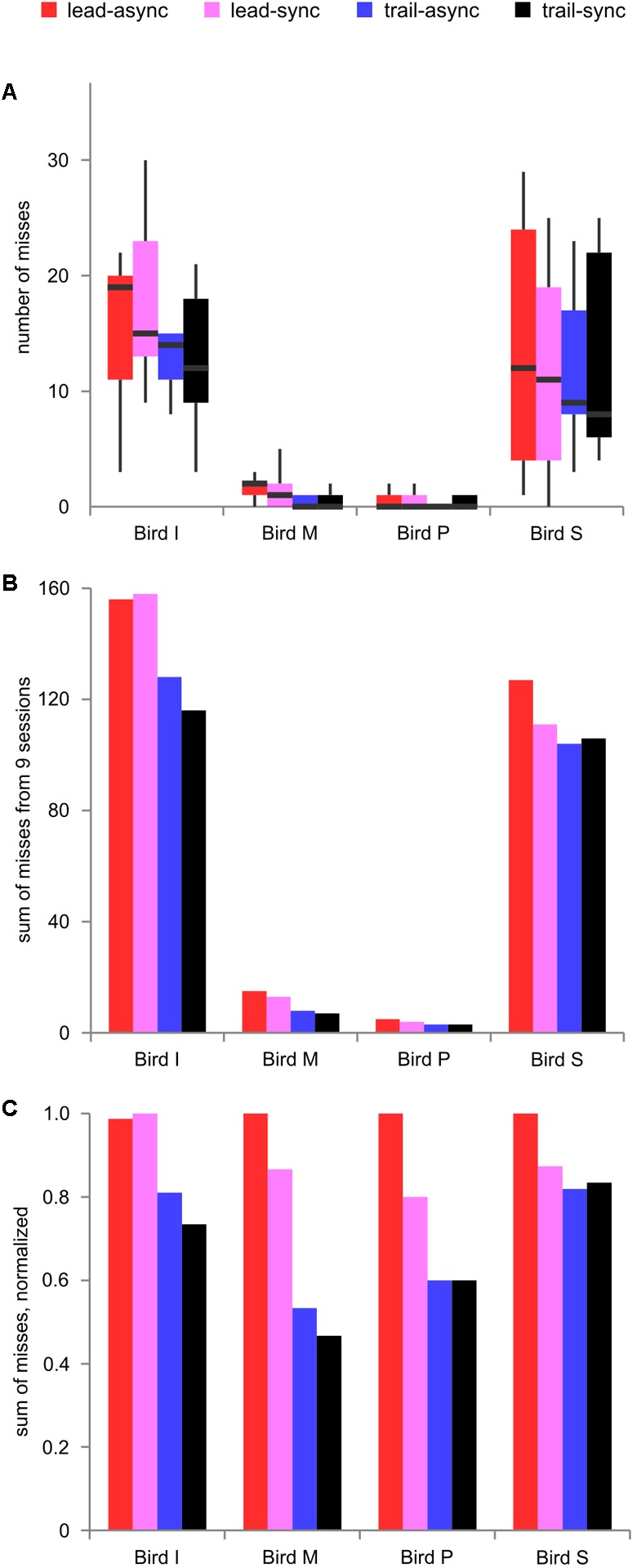
Missed trials. **(A)** Boxplot of missed trials for nine individual sessions per condition and bird, showing the variability between sessions. **(B)** Sum of missed trials across nine sessions and **(C)** normalized sum of missed trials, for each bird tested in each condition. Color coding as in **Figure 2**.

Next, the number of missed trials was analyzed to find a potential relationship between the task’s difficulty level and response suppression. The foregoing results show better performance for the leading than the trailing conditions. Because the birds greatly differed in how conservative they were during decision making, the number of misses differed largely (median 59.5, range 3–158). Thus, we show the raw data and the data normalized to the maximum count per bird in **Figure 4**. There was a significant effect of order, *F*(1,9) = 9.036, *P* = 0.0148, but no effect of timing, *F*(1,9) = 0.545, *P* = 0.479, or interaction, *F*(1,9) = 0.025, *P* = 0.878. As can be seen from **Figure 4**, the number of misses was generally lower in the trailing sessions than in the leading sessions. This shows that leading test conditions resulting in better performance evoked more misses than the trailing test conditions.

## Discussion

We tested whether violation of the unity assumption impairs performance in a vision-based TOJ task. When one singleton acoustic cue was paired with the second visual stimulus as compared to pairing with the first visual stimulus, the performance of the starlings was impaired. The timing, however, of the audio-visual pair (synchronous or with a 75 ms AV-lag offset) had no general effect but an acoustic cue elicited better performance when leading the first visual stimulus than when being presented synchronous to the first visual stimulus, whereas no timing effect for the trailing acoustic stimuli was observed. Previously, we tested starlings with pairs of acoustic and visual stimuli, thus meeting the unity assumption, with the stimuli being arranged in various timing combinations (Feenders et al., 2017). We found performance improvement when combining leading and trailing acoustic cues as compared to synchronous visual-acoustic pairs. Interestingly, while we found a clear performance improvement with a singleton leading A1, this improvement was not observed when a second acoustic cue was presented synchronous to V2. Furthermore, the TVE was larger for a trailing A2 and synchronous A1 than for a leading A1 and synchronous A2. When comparing our previous results with those from the present study, the following picture emerges: if each visual stimulus pair is presented in close temporal proximity to a pair of acoustic cues thereby meeting the unity assumption, the TVE can be evoked as discussed in Feenders et al. (2017) (i.e., a trailing second acoustic cue will drive the TVE in combination with a leading or synchronous first acoustic cue). In that condition, an alerting effect can operate in addition to the TVE. If, however, just a singleton acoustic cue is presented that does not comply with the unity assumption as in our current study, this will either activate only some alerting mechanism in case of a leading cue, or disrupt performance in case of a trailing cue. On the one hand, the alerting effect is in accordance with the findings from Los and Van der Burg (2013): when participants had to indicate whether a visual stimulus appeared to the left or to the right of the central fixation, response times were shorter when the stimulus was preceded by a visual or acoustic cue. The authors could further show that this effect was not driven by multisensory integration but rather by preparatory processes. We provide further support of this notion by demonstrating an alerting effect (driven by preparatory processes) of a singleton leading acoustic cue (see also Barrett and Krumbholz, 2012, for the prior entry effect in a TOJ task). On the other hand, the disruptive effect of the trailing acoustic cue, as we observed, shows that the TVE requires compliance with the unity assumption, i.e., a trailing sound by its own is not sufficient to evoke the TVE. This is important as Morein-Zamir et al. (2003) argued that the trailing sound is driving the TVE. However, they used stimulus paradigms that adhered to the rule of unity, i.e., each visual stimulus was always paired with an acoustic cue. Our data from the previous and the present study explore the differential role of a singleton versus a paired acoustic cue. Because the unity assumption is discussed as one of the major factors influencing the binding of multisensory cues and thus multisensory integration (see Chen and Spence, 2017 for a critical review), we suggest that the TOJ task as used in our study is a valid paradigm to evoke multisensory integration as opposed to attentional processes.

The general validity of the unity assumption for TOJ tasks is specifically interesting as, until now, evidence has mainly been gathered in humans with speech-like or synesthetic stimuli (Vatakis and Spence, 2007, 2008; Vatakis et al., 2008; Parise and Spence, 2008, 2009; speech-gesture pairs: Margiotoudi et al., 2014) and also for musical stimuli (Petrini et al., 2009; Chuen and Schutz, 2016). We want to point out, though, that in those studies (except Parise and Spence, 2008, who used a test paradigm very similar to ours) the TOJ task required the participants to judge whether the auditory or the visual stimulus appeared first in a cross-modal comparison. The resulting performance was better if the auditory and visual stimuli were congruent with respect to the participants’ experience, e.g., a video clip of a speech stimulus paired with an auditory sample of the same stimulus, as compared to non-congruent pairings (e.g., the video clip showing a speech stimulus different from the auditory sample). In our study, the acoustic and visual stimuli did not share any physical properties except the timing of appearance. Furthermore, our task could only be solved by attending to the visual stimuli because the acoustic stimuli did not carry any relevant information (this is similar to Parise and Spence, 2008). This shows that the unity assumption is effective in humans and starlings, in different versions of the TOJ task and not limited to speech-related stimuli.

Humans and songbirds both depend on the visual system. Birds rely on visual information for a wide range of behavioral patterns (see also Introduction) and have thus evolved a very specialized visual system with lateralized functions (reviewed in Zeigler and Bischof, 1993; Rogers, 2012). The temporal ventriloquism effect shows that the avian visual system, similar to humans, can be modified by acoustic information despite being a dominant sense.

**FIGURE 5:**
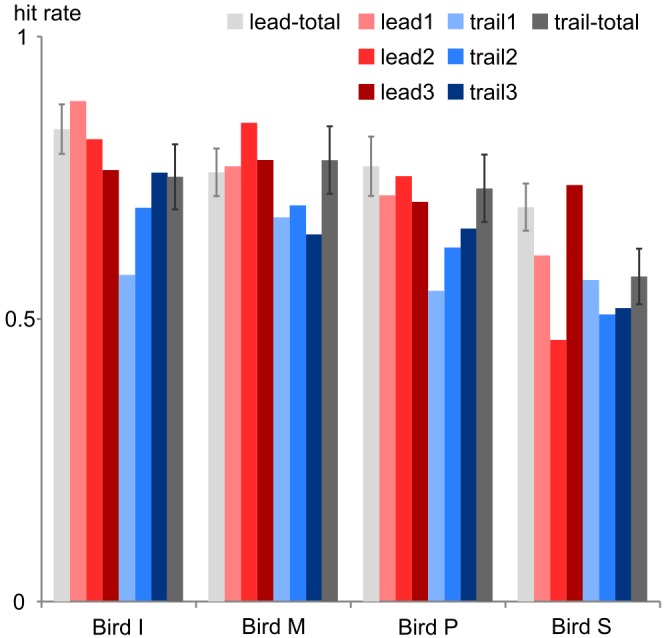
Performance change. The hit rate is shown for each bird during the first 3 leading sessions (lead1–3, red colors) and the first 3 trailing sessions (trail1-3, blue colors) and in comparison to the hit rate across all leading sessions (lead-total, light gray leftmost bars) and trailing sessions (trail-total, dark gray rightmost bars). Error bars: standard deviation.

Multisensory binding and integration is affected by experience: when spotting a cat in a tree we will mentally pair the cat with a meowing but not with the cawing of a close-by crow. Furthermore, the repeated presentation of stimuli with certain time offsets may result in temporal recalibration to reinstate synchronicity (Vroomen et al., 2004; for a review see Chen and Vroomen, 2013). Likewise, our birds seemed to adjust to the specific test conditions as we observed a drop in performance when first introducing the trailing sound condition (**Figure 5**). In all four birds, this first trailing session did not meet our criterion as a valid session. In later sessions, the overall performance increased probably due to learning and potentially recalibration effects. We kept identical conditions (either leading or trailing) for three consecutive sessions and then changed the conditions to provide enough time for adjustments to the test stimuli. By this scheme we further intended to avoid any overtraining or recalibration effects that may transfer to the other conditions if all leading conditions had been presented in one block followed by all trailing sessions or vice versa.

The difficulty of the task is higher in the trailing than the leading trials. This may also be reflected in the number of missed trials: in a (difficult) trailing trial, a decisional error might not be obvious to the bird and thus the bird will respond (incorrectly) instead of withholding the response. In contrast, in a (easy) leading trial, the bird more likely will be aware of a decisional error and may partly correct it by not responding to the incorrect target resulting in a higher number of missed trials. Indeed, we observed a higher number of missed trials in the leading than the trailing sessions. This leads us to the conclusion that missed trials are at least partly driven by a cognitive conflict between premature decisions and externally driven corrections. We want to point out, though, that a substantial number of missed trials is driven by a lack of attention as the number of missed trials did not decrease to zero in any test condition. Personal observations during the experiments support this notion with birds being clearly inattentive in some trials (averted head, preening, turning on perch and similar behavioral patterns). Based on this latter point, we believe that our earlier statement to exclude missed trials for the analysis of the hit rate is justified.

## Conclusion

In conclusion, we provide clear evidence that a singleton trailing sound in comparison to a singleton leading sound results in a decreased performance in a vision-based TOJ task. In combination with previous findings this suggests that compliance with the unity assumption is a prerequisite for multisensory stimuli to evoke the TVE. Here, the second acoustic cue will drive the TVE when presented with a lagging offset. In contrast, only a singleton acoustic cue leading the visual stimuli will cause clearly alerting effects. Our findings provide supporting evidence for a crucial role of the unity assumption in a temporal ventriloquism effect similar to the spatial ventriloquism effect.

## Author Contributions

GF designed the experiments and collected and analyzed the data. GF and GK wrote the manuscript.

## Conflict of Interest Statement

The authors declare that the research was conducted in the absence of any commercial or financial relationships that could be construed as a potential conflict of interest.

## Footnotes

1 http://www.R-project.org/
